# Supplementary material for: Systematic identification of recognition motifs for the hub protein LC8
Source: Life Sci Alliance. 2019 Jul 2;2(4):e201900366. doi: 10.26508/lsa.201900366 (PMC6607443; doi:10.26508/lsa.201900366)
Supplement: Supplementary file 1 [file LSA-2019-00366_TableS1.doc]

Supplemental Table 1 - Binding enriched peptides from proteomic peptide phage display.

| Uniprot | Gene | Peptide |
| --- | --- | --- |
| Q8WWN8 | ARAP3 | TQTPGFPTQPPATSSP |
| O43521 | BCL2L11 | DKSTQTPSPPAQAFNH |
| Q96G01 | BICD1 | KLNGDYRTPTLRKGES |
| Q92904 | DAZL | KRVHHFRRSRAMLKSV |
| Q92499 | DDX1 | TQTKFLPNAPKALIVE |
| Q96SC8 | DMRTA2 | LSSRSAFSPLQPNASH |
| Q7Z589 | EMSY | TQTTNTTTQKVIIVTT |
| Q9Y5Z7 | HCFC2 | VIPETSVSSTVSSTQT |
| Q9Y2F5 | ICE1 | ELRHIGTQISSDSYGS |
| P35568 | IRS1 | LPRKVDTAAQTNSRLA |
| O43526 | KCNQ2 | VPMYSSQTQTYGASRL |
| Q9P266 | KIAA1462 | TQTSFSEEPQSSQLLP |
| P49862 | KLK7 | STQTHVNDLMLVKLNS |
| Q8NEZ4 | KMT2C | SVSTQTASDDQAGKLW |
| Q2TV78 | MST1L | TANTTTAAYLASVGTR |
| Q02505 | MUC3A | LTSATGTQTSPAPTTV |
| Q9HC84 | MUC5B | LTSTATKSTATSVTPI |
| Q9HC84 | MUC5B | SSSTQTSGTPPSLTTT |
| Q6ZRI0 | OTOG | LTASQLPAGPTESPAS |
| Q8NDX5 | PHC3 | SSVSTTTSSTTTTTIT |
| P14859 | POU2F1 | TGTQTNGLDFQKQPVP |
| Q9ULJ8 | PPP1R9A | TDGSVVKLESSVSERI |
| Q01973 | ROR1 | SGGNATTQTTSLSASP |
| Q9H4B6 | SAV1 | FLRTPIQRTPHEIMRR |
| Q96FV2 | SCRN2 | LPRFQTQVDRRHTLYR |
| Q8TEC5 | SH3RF2 | TLVSTASGTQTVFPSK |
| Q96R06 | SPAG5 | STQTDTSHSGITNKLQ |
| Q96JG9 | ZNF469 | AAALPEETRSSRRRRL |
| Q99814 | EPAS1 | STQTDFNELDLETLAP |
| Q86VQ1 | GLCCI1 | TRSIDTQTPSVQERSS |
| P98088 | MUC5AC | VTSSITSTTQTSTTSA |
| Q2KHR3 | QSER1 | SQTVTPENQTLNYSSN |
| Q5VU36 | SPATA31A5 | GTVPQSLSPHEDLVAS |
| Q9H2K8 | TAOK3 | PSMSVSTGSQSSSVNS |
| Q6ZU65 | UBN2 | SQTNPVVKLSNNPQLS |
| Q9Y4F4 | FAM179B | QQTFGSQTEATSSNGQ |
| Q13618 | CUL3 | SGRQLTLQHHMGSADL |
| Q99102 | MUC4 | STQTTRESQTSTLTHR |
| Q9P2G1 | ANKIB1 | SSQTPQTSSDWLEQVH |
| P07359 | GP1BA | TATQTTHLELQRGRQV |
| Q9HC84 | MUC5B | LTSTATKSTATSFTPI |
| Q02817 | MUC2 | GTQTPTTTPITTTTTV |
| Q9BWV3 | CIZ1 | VSTQTGSMTGQIPRLS |
| Q8NBH2 | KY | TSYNSQGTQLTVEVHP |
| O75665 | OFD1 | SANMETQTSSTFNRDS |
| Q7Z2Z2 | EFTUD1 | TGSQTFDSFPPETQAL |
| A0FGR9 | ESYT3 | VSRSTTTTTSATTVAT |
| Q13952 | NFYC | ITQTEVQQGQQQFSQF |
| Q9Y4K1 | AIM1 | SFVLPVESTQDVSSQV |
| Q6IMN6 | CAPRIN2 | STQTPPQCQLPSIHVE |
| P53350 | PLK1 | LQTDPTARPTINELLN |
| Q9UBY0 | SL9A | GTQTSGLLQQPLLSKD |
| P48436 | SOX9 | SQYDYTDHQNSSSYYS |

^a^ Peptides identified from next generation sequencing of binding enriched phage pools as well as peptides identified by ELISA experiments are shown.

Phage display hits validated via ITC are highlighted in grey.

Motif anchors are underlined.
