# Supplementary material for: Systematic identification of recognition motifs for the hub protein LC8
Source: Life Sci Alliance. 2019 Jul 2;2(4):e201900366. doi: 10.26508/lsa.201900366 (PMC6607443; doi:10.26508/lsa.201900366)
Supplement: Supplementary file 3 [file LSA-2019-00366_TableS3.doc]

Supplemental Table 3 - LC8 binding sequences identified and verified in this study.

| UniProt | Gene | 8AA Sequence |  |
| --- | --- | --- | --- |
| Q6LCS3 | E4 (HPV) | EDKQTQTP | |
| O43521 | BCL2L11* | CDKSTQTP | |
| O43719 | HTATSF1 | KDGDTQTD | |
| O75665 | OFD1* | CNMETQTS | |
| P07359 | GP1BA | LTTATQTT | |
| P11193 | VP4 (Rotavirus A) | NDISTQTS | |
| P13500 | CCL2 | LDKQTQTP | |
| P16289 | L (RAV)*^a^ | ISRMTQTP | |
| P18583 | SON | RCVSVQTD | |
| P80098 | CCL7 | LDKKTQTP | |
| Q01973 | ROR1 | GNATTQTT | |
| Q02505 | MUC3A | SATGTQTS | |
| Q05127 | VP35 (EBV)*^a^ | RNSQTQTD | |
| Q6IMN6 | CAPRIN2 | TTASTQTP | |
| Q86VQ1 | GLCCI1 | RSIDTQTP | |
| Q8IX07 | ZFPM1 | SDKGVQTP | |
| Q8N4C6 | NIN | RTSETNTP | |
| Q8WWN8 | ARAP3 | TGLPTQTP | |
| Q96R06 | SPAG5* | QDSSTQTD | |
| Q99102 | MUC4 | WTRSTQTT | |
| Q9HC10 | OTOF | SSTEVQVE | |
| Q9UBY0 | SLC9A2 | REKGTQTS | |
| Q9ULV3 | CIZ1* | RSVSTQTG | |
| Q9UPA5 | BSN^II^* | AEFSTQTP | |
| Q9UPA5 | BSN*^a^ | ANYGSQTE | |
| Q9UPA5 | BSN^III^*^a^ | VAQGTQTP | |
| Q9Y2F5 | ICE1 | RHIGTQIS | |
| Q9Y2H9 | MAST1* | RHQSVQTE | |
| Q9Y4F4 | TOGARAM1 | QTFGSQTE | |

^a^ Peptides synthesized to validate previous data, not based on predictions

Motif anchors are underlined.
