# Supplementary material for: Systematic identification of recognition motifs for the hub protein LC8
Source: Life Sci Alliance. 2019 Jul 2;2(4):e201900366. doi: 10.26508/lsa.201900366 (PMC6607443; doi:10.26508/lsa.201900366)

**Supplemental Information:**

**Improved Algorithm Generation**

We aligned 79 experimentally confirmed LC8 binding sequences (Table S4). Only ‘strong’ binding sequences (*K_d_* < 25 μM) were selected so that the final algorithm provided only high-confidence hits, rather than being exceptionally permissive; however, this means that many weak binding sequences will be overlooked. Aligned motifs were defined as an eight amino-acid peptide sequence$p$, stretching from the -5 to the +2 positions relative to the anchor Q residue, thereby defining the “0” position of each binding sequence as the Q in the canonical TQT motif. We used these binding sequences to generate a PSSM with weights, $W_{ai}^{B}$, for a given amino acid, $a$, at position, $i$, such that $p[i]=a$ within each instance of the motif. These weights (eq. 1) define a log-likelihood score comparing the frequency of the amino acid at that position in the binders, $B_{ai}$, to the background frequency of amino acid, $P_{a}$, across disordered eukaryotic proteins found in DisProt, a database of intrinsically disordered proteins and regions (Piovesan et al., 2017). A positive weight at a given position and amino acid indicates that that amino acid is enriched at that position, relative to a random occurrence rate. Likewise, a negative weight indicates that the amino acid is depleted at that residue.

$W_{ai}^{B}=\log_{2}\left( \frac{B_{ai}}{P_{a}} \right)$ eq. 1

$B_{ai}$ is taken as the number of counts $C_{ai}$ plus a pseudo-count$D,$over the total number of counts at position $i$. Pseudo-counting was primarily used to fill gaps in the matrix, and a value of 5 was selected for $D$ based on prior work in the field (eq. 2; Erdős et al., 2017).

$B_{ai}=\frac{C_{ai} + \frac{D}{20}}{D + \sum_{a=1}^{20} C_{ai}}$ eq. 2

The frequency matrix $B_{ai}$ is then used to compute $W_{ai}^{B}$ as in eq. 1. In addition to a matrix informed by known binding sequences, we assembled a matrix normalized to nonbinding sequences, each determined via ITC to not bind to LC8. We aligned 32 anchor-containing sequences in the same manner as above and used them to generate a PSSM with weights $W_{ai}^{N},$incorporating both binding and nonbinding sequences. For this matrix, instead of the background frequency of a given amino acid, the frequency within the binders was normalized to the frequency $N_{ai}$of the same amino acid in the non-binders (eq. 3).

$W_{ai}^{N}=\log_{2}\left( \frac{B_{ai}}{N_{ai}} \right)$ eq. 3

*N_ai_* was assembled in the same manner as $B_{ai}$, using non-binding sequences, and the resultant PSSM provides log-scale scores that reflect relative enrichments for each amino acid at a given position in the known binding sequences, versus in the known non-binding sequences. A positive$W_{ai}^{N}$ value corresponds to an amino acid at a given position being relatively enriched in the binding sequences, while a negative value corresponds to it being enriched in non-binding sequences.

To further improve our ability to differentiate binders and non-binders, we built additional PSSMs that binned amino acids into four categories. First, PSSMs were assembled based on properties of charge and polarity, with a bin for positively charged (H,K,R), negatively charged (D,E), polar (C,N,Q,S,T,W,Y), and hydrophobic amino acids (A,F,G,I,L,M,P,V). In addition, another set of matrices was assembled based on amino acid volume, also with four bins: very small: less than 106 Å^3^ (A,C,G,S); small: 122 to 142 Å^3^ (D,N,P,T,V); medium: 155 to 171 Å^3^ (E,H,I,K,L,M,Q); and large: greater than 200 Å^3^ (F,R,W,Y; Richards, 1977). Groupings for amino acid volume were chosen to minimize the range of volumes within a given group.

In this manner, a total of 6 PSSMs were constructed, two each corresponding to amino acid bins, polarity/charge based bins, and volume based bins. For a given input sequence, each matrix returns a score, equivalent to the sum of the weights at the relevant amino acids and position (eq. 4). For clarity, each matrix was given a subscript, $S_{B}$ for the matrices built using only binding sequences, and $S_{N}$ for matrices using both binder and non-binder data. The polarity and charge matrices were indicated with a P ($S_{PB}, S_{PN}$), and the volume matrices were indicated with a V ($S_{VB}, S_{VN})$

$S(p)= \sum_{i=-5}^{2} W_{P\left[ i \right],i}$ eq. 4

To simplify the output of our scoring system, we simplified our output to two scores. The first metric used the first two matrices described, which scored input peptides on their amino acid sequence (eq. 6). The resultant score, $S_{aa}(p)$, is the sum of the scores for both the binder-only matrix $W_{ai}^{B}$ and the non-binder normalized matrix $W_{ai}^{N}$ across all positions $i$ in the motif. Both scores are modified by weight factors ($\alpha_{B},\alpha_{N}$, respectively) that were optimized using a ROC curve computed using leave-one-out cross validation, whereby a separate set of weights $W_{p\left[ i \right],i}^{B},W_{p\left[ i \right],i}^{N}$was calculated at the exclusion of each peptide.

$S_{aa}(p)= \sum_{i=-5}^{2} {(\alpha}_{B}W_{p\left[ i \right],i}^{B}+\alpha_{N}W_{p\left[ i \right],i}^{N})$ eq. 5

The second metric scored sequences based on their volume and polarity properties, using values from our four additional PSSMS score (eq. 7). The score $S_{vp}$(*p*) combines scores from a background frequency normalized matrix with polarity bins $W_{ai}^{PB}$, a non-binder normalized matrix with polarity bins $W_{ai}^{PN}$, as well as equivalent matrices using volume bins $W_{ai}^{VB},W_{ai}^{VN}$. These were also modified by weight factors, $\alpha_{PB}$, $\alpha_{PN}$, $\alpha_{VB}$, $\alpha_{VN}$.

$S_{VP}(p)= \sum_{i=-5}^{2} {(\alpha}_{PB}W_{p\left[ i \right],i}^{PB}+\alpha_{PN}W_{p\left[ i \right],i}^{PN}+\alpha_{VB}W_{p\left[ i \right],i}^{VB}+\alpha_{VN}W_{p\left[ i \right],i}^{VN})$ eq.6

To test our approach, we scored our training sequences using leave-one-out cross validation, where one sequence was omitted from the training set used to generate each scoring matrix. The omitted sequence was then scored on the PSSMs generated, with this process repeated for every peptide used. Scoring was evaluated via an ROC curve. The area under the ROC curve (AUROC) corresponds with the score’s ability to separate true positives – sequences which do bind LC8 and which also score as binders – from false positives – sequences that do not bind, but still return a positive score. The larger the AUROC, the greater the ability of the score to differentiate binding from non-binding sequences (Supplemental Figure 1). Using the AUROC as a metric for PSSM effectiveness, we did a grid search of possible weight factor values for equations 5 and 6. The arrived at $\alpha$values are as follows: $\alpha_{B}$= 1, $\alpha_{N}=0.78$, $\alpha_{PB}=0, \alpha_{PN}=0.6$, $\alpha_{VB}$ = 0.37, $\alpha_{VN}$ = 0.22. Notably, the settled on $\alpha$for the polarity matrix that used only binding sequences was optimized to 0, indicating that the binder-only polarity matrix did not improve the ability of $S_{VP}$ to separate binding and non-binding sequences.

Additionally, we used the AUROC to determine that the non-binder normalized scores $W_{ai}^{N}, W_{ai}^{PN}, W_{ai}^{VN}$ had a negative impact on the effectiveness of our scoring when included at the -1, 0 and +1 position in the matrices. As the non-binding sequences were selected for the presence of a TQT anchor, there is effectively no relative enrichment for any amino acid or physiochemical property at those positions in the associated PSSMs, and we therefore excluded those scores at the positions in question.

**Citations**

Erdős, G., Szaniszló, T., Pajkos, M., Hajdu-Soltész, B., Kiss, B., Pál, G., … Dosztányi, Z. (2017). Novel linear motif filtering protocol reveals the role of the LC8 dynein light chain in the Hippo pathway. *PLoS Computational Biology*, *13*(12), e1005885. https://doi.org/10.1371/journal.pcbi.1005885

Piovesan, D., Tabaro, F., Mičetić, I., Necci, M., Quaglia, F., Oldfield, C. J., … Tosatto, S. C. E. (2017). DisProt 7.0: a major update of the database of disordered proteins. *Nucleic Acids Research*, *45*(D1), D219–D227. https://doi.org/10.1093/nar/gkw1056

Richards, F. M. (1977). Areas, Volumes, Packing, and Protein Structure. *Annual Review of Biophysics and Bioengineering*, *6*(1), 151–176. https://doi.org/10.1146/annurev.bb.06.060177.001055

**Supplemental Figure 1 – Optimization of matrix weights. (A)** ROC curves of both amino acid matrices and S_aa_. The larger the area under the curve (AUROC), the more effective the curve is at separating binder sequences from non-binder sequences. **(B)** ROC curves of each volume and polarity matrix, and S_vp_. Notably, the S_vp_ curve performed substantially better than each volume and polarity matrix individually, which suggests that volume and polarity are both essential to understanding the preferences within the LC8 motif.


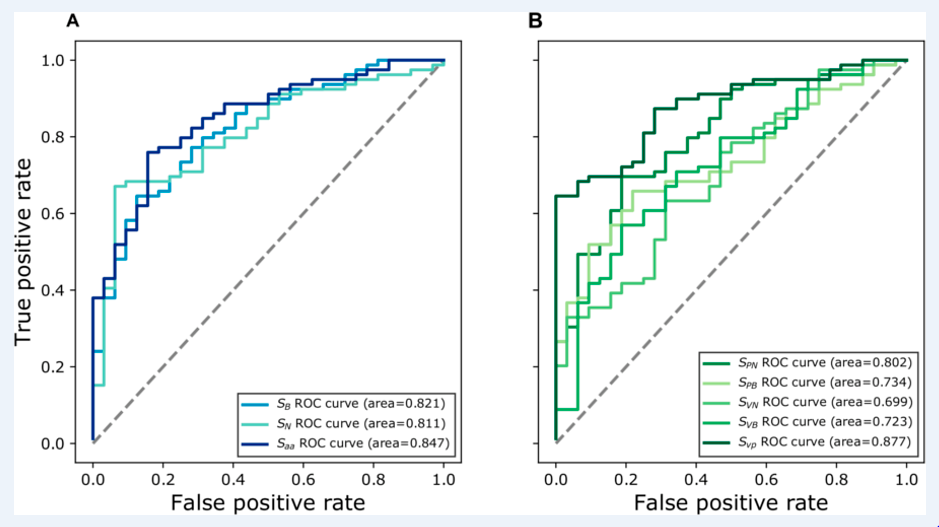

Supplement: Supplementary file 5 [file LSA-2019-00366_Supplemental_Data_1.docx]
